# Using 3D CityGML for the Modeling of the Food Waste and Wastewater Generation—A Case Study for the City of Montréal

**DOI:** 10.3389/fdata.2021.662011

**Published:** 2021-06-22

**Authors:** Reiner Braun, Rushikesh Padsala, Tahereh Malmir, Soufia Mohammadi, Ursula Eicker

**Affiliations:** ^1^ Canada Excellence Research Chair Next Generation Cities, Gina Cody School of Engineering and Computer Science, Concordia University, Montréal, QC, Canada; ^2^ Herman Hollerith Center (HHZ), Faculty of Informatics, Reutlingen University, Böblingen, Germany; ^3^ Centre for Geodesy and Geoinformatics, University of Applied Sciences Stuttgart, Stuttgart, Germany

**Keywords:** CityGML, data model, food waste, wastewater, anaerobic digestion, key performance indicators, FEW nexus

## Abstract

The paper explains a workflow to simulate the food energy water (FEW) nexus for an urban district combining various data sources like 3D city models, particularly the City Geography Markup Language (CityGML) data model from the Open Geospatial Consortium, Open StreetMap and Census data. A long term vision is to extend the CityGML data model by developing a FEW Application Domain Extension (FEW ADE) to support future FEW simulation workflows such as the one explained in this paper. Together with the mentioned simulation workflow, this paper also identifies some necessary FEW related parameters for the future development of a FEW ADE. Furthermore, relevant key performance indicators are investigated, and the relevant datasets necessary to calculate these indicators are studied. Finally, different calculations are performed for the downtown borough Ville-Marie in the city of Montréal (Canada) for the domains of food waste (FW) and wastewater (WW) generation. For this study, a workflow is developed to calculate the energy generation from anaerobic digestion of FW and WW. In the first step, the data collection and preparation was done. Here relevant data for georeferencing, data for model set-up, and data for creating the required usage libraries, like food waste and wastewater generation per person, were collected. The next step was the data integration and calculation of the relevant parameters, and lastly, the results were visualized for analysis purposes. As a use case to support such calculations, the CityGML level of detail two model of Montréal is enriched with information such as building functions and building usages from OpenStreetMap. The calculation of the total residents based on the CityGML model as the main input for Ville-Marie results in a population of 72,606. The statistical value for 2016 was 89,170, which corresponds to a deviation of 15.3%. The energy recovery potential of FW is about 24,024 GJ/year, and that of wastewater is about 1,629 GJ/year, adding up to 25,653 GJ/year. Relating values to the calculated number of inhabitants in Ville-Marie results in 330.9 kWh/year for FW and 22.4 kWh/year for wastewater, respectively.

## Introduction

Urbanization and economic growth have increased energy use worldwide. Fossil fuels such as coal, oil, and natural gas have been the primary energy resources in various industries, even though they lead to a considerable increase in greenhouse gas (GHG) emissions. The European Union has ambitious goals to tackle climate and environmental-related challenges; with its Green Deal strategy, the European Union aims to be the first climate-neutral continent (European Commission 2019). Environmental challenges like climate change and the loss of biodiversity are global problems and cannot be solved by only a few actors. The population shift to cities provides unprecedented difficulties when considering food, energy, and water delivery to the urban population and the amount of waste produced from these domains. To support formulating a sustainable climate change adaptation and mitigation strategy, decision-makers such as governments, investors, and city developers must understand, quantify, and visualize multiple interdependent impacts of the food, energy, and water (FEW) infrastructures and their nexus. Looking at the increase in publications related to the FEW nexus in the past years, it becomes clear that research on the FEW nexus is a fast-growing, multidisciplinary, and inter-sectoral research area. With the Bonn Conference in 2011, FEW nexus topics moved into the scientific and non-scientific focus (Hoffman 2011). Although cities can provide services more efficiently than rural areas, many services in cities are still very resource-intensive and need to be optimized in many ways. The GHG protocol outlines how emissions can be accounted for. The protocol distinguishes between direct and indirect sources of emissions. Direct emissions are classified as scope 1 and occur at facilities directly owned or controlled by the reporting entity. Indirect emissions result from facilities owned or controlled by another company but are part of whose emissions result from the reporting entities’ activities. Indirect sources are either scope 2 or scope 3. Following the GHG protocol, the accounting in a city can be approached similarly to the accounting of a company’s emissions, with all emissions generated within the city boundaries falling under scope 1. In the following paper, only food waste (FW) and wastewater that fall into scope 1 are considered; food production usually does not occur within the city boundaries and is not considered. The world produces 2.01 billion tons of waste annually, and waste to energy technologies provide approximately 1.5% of the final energy consumption in Europe (Mayer et al., 2019). The European Landfill Directive in 1999 marked a policy to prevent the landfilling of organic waste (OW). It required members to reduce the quantity of biodegradable municipal waste sent to landfills to 75% (2006), 50% (2009), and then 35% (2016) compared to 1995 (Evangelisti et al., 2014). OW, an excellent energy source, reaches around 50–70% of the total waste produced in low and middle-income settings, contrasting the 20–40% for high-income households. It is possible to considerably reduce the methane (CH_4_) emissions by using composting or other OW treatment options (Mertenat et al., 2019). Different strategic plans for waste diversion from landfills were recently developed to increase energy generation and material recovery from waste. Sustainable Montréal 2016–2020 plan aimed at banning the disposal of OW and reaching 60% diversion from landfill. However, this target could not be achieved, and the deadline was postponed to 2030. In 2017, OW accounted for 369 kt, from which around 23% was recovered. Especially in the nexus approach, it is important to examine areas that have overlapping effects. One link between FW and wastewater is the energy sector. In both areas of FW and wastewater, organic substances are produced from which methane (energy) can be obtained. Wastewater treatment facilities are one of the significant energy users at the municipal level worldwide. Estimates represent that these facilities may require about 1–3% of a country's total electric energy output on average. The power consumption of state-of-the-art wastewater treatment facilities should range between 20, and 45 kWh per population-equivalent served per year. However, older plants may have even higher usage (Capodaglio and Olsson 2020). For instance, a wastewater treatment plant in Rzeszów, Poland, in the year 2016, treated about 42,631 m^3^/day wastewater. The average energy consumption of this plant amounted to 0.468, 0.397, and 0.865 kWh/m^3^ for electricity, heat energy, and total energy usage indicator, respectively (Masłoń 2017). Hernández-Sancho et al. (2011) compared the average electric consumption indicator of other countries: in United States 0.45 kWh/m^3^, in Switzerland 0.52 kWh/m^3^, in Spain 0.53 kWh/m^3^, in Singapore 0.56 kWh/m^3^, in the United Kingdom 0.64 kWh/m^3^ and 0.67 kWh/m^3^ in Germany (Hernández-Sancho et al., 2011). Hence, wastewater treatment plants play a crucial role in FEW nexus topics.

New simulation and data analytics tools to manage and analyze large and heterogeneous urban data sets from very different domains are needed. In this respect, an integrated urban data analysis and modeling platform is an essential software infrastructure for smart, sustainable, and resilient city planning, operation, and maintenance (Eicker et al., 2020). As a backbone to the urban data part of the platform, an integrated FEW data model is very critical. In the last decade, 3D city models, particularly the City Geography Markup Language (CityGML) from the Open Geospatial Consortium, have gained much popularity. The open data model of CityGML allows spatial modeling of semantically different georeferenced objects such as buildings and other physical elements of the real world. These CityGML data models can also be further extended to accommodate domain-specific objects and attributes along with their visualisation capacities. Future research should investigate how CityGML models can be applied to specific implementation cases such as FW and wastewater. This paper focuses on the following three research questions (RQ):

RQ1: What are the current hurdles that need to be overcome to use a CityGML data model as a basis for a city-scale automated analysis of the FEW nexus?

RQ2: What public data sources can be used as input for simulation models and corresponding libraries?

RQ3: Where are the uncertainties, errors and which parameters are important to reduce the gap between the simulated and measured values?

This paper investigates how a concept for a CityGML-based FEW data model can look like to answer the research questions. Furthermore, to develop a FEW data model, relevant key performance indicators are investigated, and the relevant datasets necessary to calculate and integrate these indicators into an urban data and modeling platform is studied. Finally, different calculations are performed for the downtown borough Ville-Marie in the city of Montréal (Canada) for the domains of FW and wastewater generation.

## Key Performance Indicators Relevant for This Study

To address sustainable resource use, human well-being, and equity, as well as integrated assessments of water, energy, and food sectors, new nexus indicators are required (Hoffman 2011). The calculation of the right indicators can help understand, analyze and quantify the nexus between two or more sectors. But which are the right indicators to evaluate the nexus on different scales? A literature study was carried out analyzing relevant indicators used to assess the impact of measures taken in each domain and its impact on the other domains. Arthur et al. (2019) analyzed the trend of indicators used in the urban nexus system and the relations to other independent factors, such as climate. A total of 226 indicators were compiled in the study and classified into the three main categories: fluxes, efficiency, and environmental impact indicators. The use of indicators enables specific assessment in individual management domains and analyzes the individual flows of resources and their usage within the urban system to assess their security and sustainability. Flux indicators analyze the individual input and output flows of resources within a given system. The efficiency indicators can evaluate the resource use and the performance per unit inflow in a system (input and outcome generated). Finally, the environmental indicators can help analyze the environmental and health impacts associated with the production and the consumption of resources within the urban system. Examples of environmental indicators are GHG emission, solid and liquid waste associated with food, water, and energy resources. In this study, the same structure as proposed by Arthur et al. (2019) is used to classify the indicators for the domains of waste-to-energy and water-to-energy (see Table 1). Additional to the indicator type and to which domains the indicator connects, a short description and the units to calculate the indicator is given. Indicators connecting all three domains are the most interesting ones, and the calculation of these indicators should be prioritized.

**TABLE 1:** Relevant indicators in the Food (F), Energy (E) and Water (W) domain identified for this study.

Description	Type	Related-to	Unit
Share of FW in total food consumption per capita	Eff	F	%
Share of wastewater generation in (fresh) water consumption per capita	Eff	W	%
Generation of FW per capita, patient, employee, or student	Flux	F	m^3^/(capita*year)
Generation of wastewater per capita, patient, employee, or student	Flux	W	m^3^/(capita*year)
Energy recovered from anaerobic digestion of FW	Flux	F-E	GJ/year
Energy recovered from anaerobic digestion of wastewater	Flux	W-E	GJ/year
Energy recovered from anaerobic co-digestion of FW and wastewater	Flux	F-E-W	GJ/year
Savings in greenhouse gas emission (GHG)	Environmental	F-E-W	tons/year

The indicators proposed in Table 1 are used to compare the performance of the case study area of Ville-Marie with values from literature and other official data sources.

## Methodology

**FIGURE 1:**
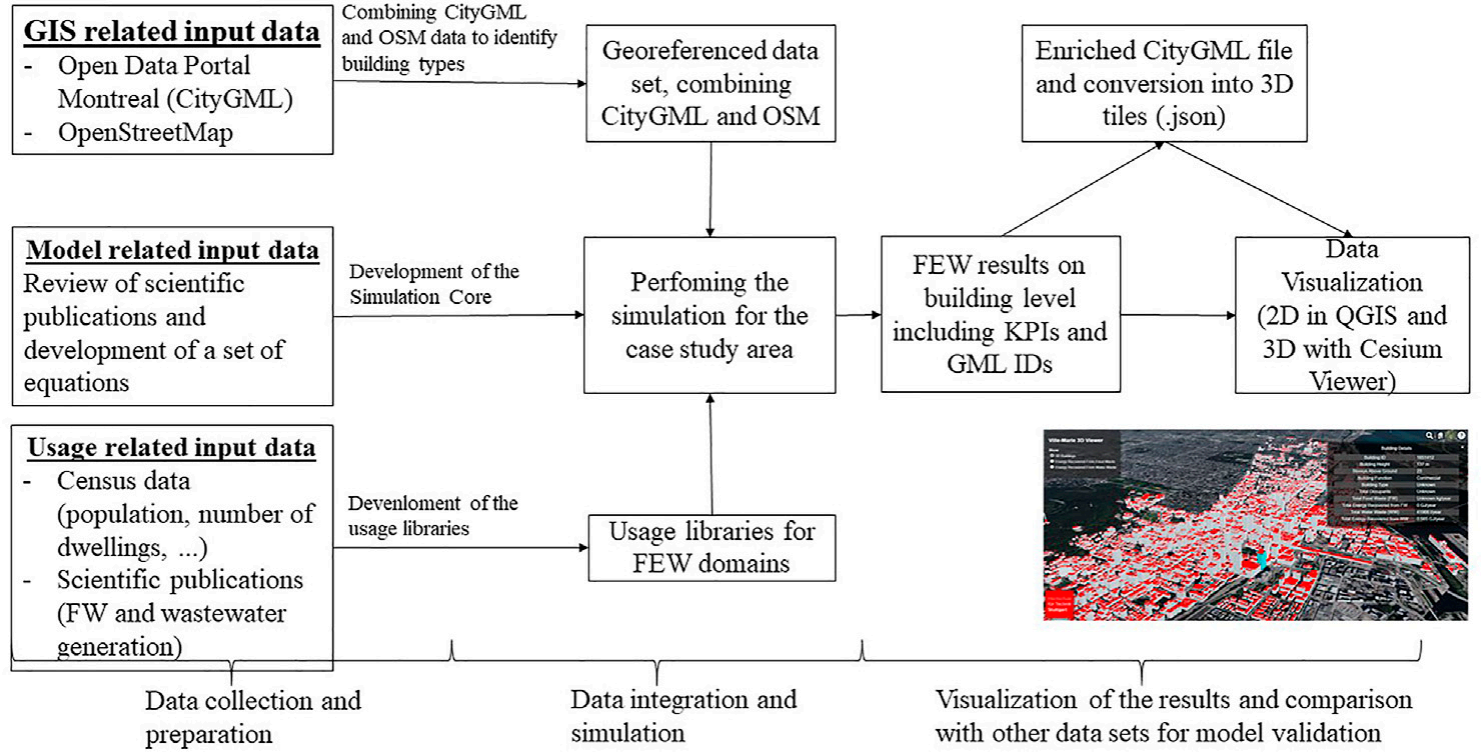
The workflow developed for this study.

For the consideration of the interrelations in the FEW nexus, on the one hand, physical or mathematical simulation models are needed to calculate the indicators, such as the energy recovered from anaerobic digestion. To structure the input data and parameters of the models, corresponding data models for the simulation are also necessary. In the following section, state of the art for simulation models and tools are presented. Further, potential attributes of the FEW data model that could be used for the development of a FEW application domain extension (ADE) are proposed, which can be used as input for different simulations. Figure 1 shows the workflow of this study. In the first step, the data collection and preparation was done. Here relevant data for model set-up, and data for creating the required usage libraries (like food waste and wastewater generation per person, see parameters in Table 8 in the Supplementary Material) were collected. The next step was the data integration and calculation of the relevant parameters, and lastly, the results were visualized for analysis purposes.

### Data Modeling of Food Energy Water Systems and its Integration Into Simulation Platforms

To analyze the complex cross-scale connections/intersections of the FEW domains, new simulation and data analytic tools need to be developed. The modeling of a system can be done in different ways. When modeling systems, it is essential to consider the question the model has to answer. The following challenges arise when creating a simulation model: 1) should the results of the simulations be visualized and who uses the visualizations, 2) which level (micro- or macroscopic) should be considered, and 3) should the simulations have a spatial and temporal dimension. Quantitative models like system dynamics (SD) models (e.g., causal loop diagrams to analyze supply chains) on the one hand and analytical models and mathematical programming, simulation methods, on the other hand, are suitable for the modeling of systems. Key elements in SD models are the modeling of feedback and delay mechanisms. The first step in mapping the system structure and links between the elements of the system structure is to develop a causal diagram, which illustrates the cause-and-effect relationship between the connected system elements. SD modeling is suitable to simulate and examine complex and dynamic systems to support long-term, strategic decision-making (Rebs et al., 2019). SD models can therefore be used to investigate the impact of new interventions. For any simulation platform, a robust data model is a must. The present work focuses on extending the open data model of CityGML further with FEW specific parameters. In general, there are two ways to develop the data model of CityGML: 1) generics and 2) application domain extension (ADE). Both these methods are CityGML inbuilt mechanism to augment its data model with application-specific geometries and/or attributes. While generics are the easiest way to expand the CityGML data model by introducing user-defined city objects (geometries) and attributes, it must be used with utmost care and only if there is no appropriate thematic CityGML class (e.g. building, landuse, waterbody, road etc.) available in the overall CityGML schema. Otherwise, issues concerning schema validation and semantic interoperability may arise. Bao et al. (2020) demonstrated a CityGML based biomass workflow applied on two different German counties wherein the food energy nexus was simulated to evaluate the local biomass potential and its transformation to different forms of biofuel with their thermal and electrical energy potential. Such a workflow was made possible by extending the landuse thematic class of CityGML using generic attributes of local crop type and landuse area. This workflow was extended by Bao et al. (2020), further enriching the CityGML data model of land use with a generic attribute of local soil type to estimate the impact on the water demand by the local bio-energy potential. While generics do not change the XML schema of CityGML, ADEs must be formally specified in the XML schema. ADEs have their namespace to prevent conflicts with other CityGML thematic classes hence issues with semantic interoperability in the case of generics are no longer a bottleneck. According to the latest overview on ADE developments made by Biljecki et al. (2018), over 44 ADEs are supporting different application domains. A few notable ADEs are available via the home page of CityGML (http://www.citygmlwiki.org/index.php/CityGML-ADEs). In particular, the Energy ADE (Nouvel et al., 2015) comes close to the present context of the paper. The Energy ADE is the first of its kind CityGML ADE which supports large-scale urban energy modeling for building stocks. It is applied for calculating energy demand on a broader spatial extent such as neighborhoods, districts, or cities. Furthermore, the XML structure opens possibilities for data exchange between different tools, users, and stakeholders. As of today, the Energy ADE is supported by a number of urban energy simulators such as SimStadt (Nouvel et al., 2015a, Nouvel et al., 2015b), TEASER+ (Avichal Malhotra et al., 2019), CitySimPro (Rosser et al., 2019), EnergyPlus (Lilis et al., 2016) or the Ladybug tools of grasshopper for Rhinoceros3D (Wang 2020). Many use cases such as the city of Helsinki in Finland (Rossknecht and Airaksinen 2020), the County of Ludwigsburg in Baden-Wuerttemberg, Germany (Bruse et al., 2015), the district of Meidling in Vienna, Austria (Agugiaro 2016) have successfully demonstrated the use of Energy ADE along with urban energy simulators to assess their building stock energy demand. Although with the Energy ADE, the building stock energy demand can be modelled, a data model that can also model synergies between food, energy, and water is still missing at the time of writing (January 2021). Such a FEW data model could support effective strategies to combat climate change adaptation and mitigation. It would help to move out of individual domain silos and evaluate the built environment as a whole to understand the synergies of different domains. Within this paper, a first attempt to develop an inclusive CityGML based FEW data model is proposed, which can be further used to calculate and web geo-visualize the indicators as mentioned in Table 1. Since at present no urban energy simulator supports such FEW based simulations, the programming platform MATLAB is used for the implementation and calculation. As a use case to support such calculations, the CityGML level of detail two model of Montréal is enriched with information such as building functions and building usages from OpenStreetMap—https://www.openstreetmap.org/ (OSM) data and occupancy details based on Census publications.

Table 2 represents the first collection of potential attributes which should be considered for the future development of a FEW ADE. Parameters mentioned in point 1) can be directly calculated from the input semantic building geometries. Parameter mentioned in point 2) can be added as an ADE element to the existing abstract class “*_AbstractBuilding*” of the CityGML building data model. Parameters in point 3), 4) and 5) are inherited from the “*_AbstractBuilding*” class according to its building use attribute and are new classes specific to the food, water and energy domain, respectively. However, as already mentioned in this paper, only the FW calculations, wastewater generation, and their energy recovery potential are further investigated.

**TABLE 2:** Potential attributes of the FEW data model that could be used for the development of a FEW ADE.

No	Description	Attributes
A	Potential parameters that can directly be calculated using the semantic data model of CityGML	Building envelope area (ground, envelope, roof), building volume, and building height, floor height
B	Potential parameters that can be added to the *“_AbstractBuilding”* class of CityGML building data model—ADE element	Number of occupants, actual usable floor space for the building usage
C	Potential parameters that can be assigned to in food domain—new class in the FEW ADE	Residential—food production and consumption, food resources
		Commercial—Waste generation intensity
D	Potential parameters that can be assigned to in water domain—new class in the FEW ADE	Residential—water consumption per capita, household water consumption is classified into different end-uses: showering, bathing, hand wash basin tap use, toilet flushing, dishwashing, clothes washing, cooking, house floor washing, vehicle washing, garden watering, and swimming pool. Black, grey and green water
		Commercial—Contaminated water generation (e.g. through medicals in hospitals)
		Industrial—Industrial process water consumption
E	Potential parameters that can be assigned to in energy domain - new class in the FEW ADE	Energy used for pumping of water, energy yield from FW, total energy consumption per wastewater treated, fossil fuel consumption, electricity consumption per capita, hydropower potential, groundwater energy

**FIGURE 2:**
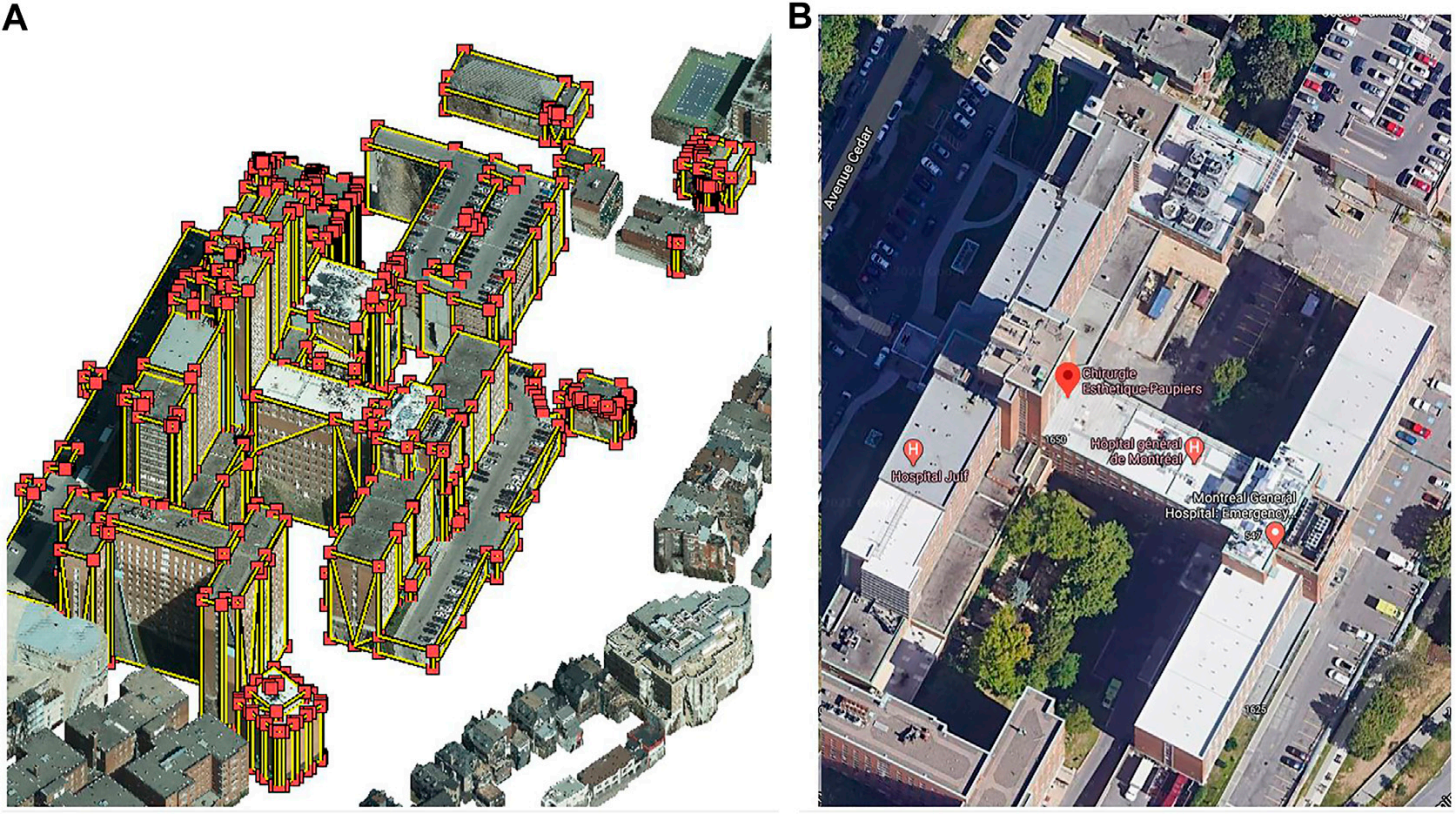
3D CityGML model of a hospital building in Ville-Marie **(A)** and Google Street Map view **(B)**.

To support FW and wastewater related calculations, an important parameter is building occupants. By combining the individual data sources (CityGML, OSM), it is possible to estimate how many people *P* live in a residential building, work in a hospital, or how many students visit a school. For this purpose, an expected number of stories per building type is first calculated from the building height; the number of stories is obtained by dividing the building height by a typical floor height of the building type.

$$P=\frac{h_{GML}}{h_{f}}\times A_{gr}\times f_{usage}\times A_{P}\times R_{cube,GML},$$ (1)

where *h*_*GML*_ is the building height taken from the CityGML model, *h*_*f*_ the typical floor height for each building type (residential, commercial, and industrial) $\left(h_{GML}/h_{f}\right)=N_{f}\;\text{number}\;\text{of}\;\text{floors}$, *A*_*gr*_ the ground area (building footprint) of the building from the CityGML model, *f*_*usage*_ is a factor that takes into account areas such as staircases or elevator shafts that are included in building ground area but cannot be counted towards the actual usable floor space, in this study, a constant value of 0.75 was taken into account for *f*_*usage*_, and *A*_*P*_ the average floor area used per group (resident, employee, student, etc.) in that region in m^2^. The factor *R*_*GML*_ takes into account the ratio of building envelope area to building volume, which can be extracted from 3D models (see Figure 2 and Eq. 1 in Supplementary Material). *R*_*cube,GML*_ is introduced to avoid an overestimation of the total floor area. To derive the number of floors per building type, the building height from the CityGML model is used and combined with the calculated floor height per building type (see Table 2 in the Supplementary Material).

Additionally, the OSM tags building:height and building:levels could also be used to get information on the building height and the number of floors per building. The OSM data set for Quebec downloaded from Geofabrik Server did not provide the required information on the height and floors per building, so it was not possible to include this data into our workflow.

Since this paper aims to show the first attempt of a CityGML based FEW simulation workflow, a generic model of extending the CityGML data model of building is used to save time and complexity. This data model is foreseen as the first step in developing a CityGML FEW ADE.

## Case Study City of Montréal—Borough Ville-Marie

**FIGURE 3:**
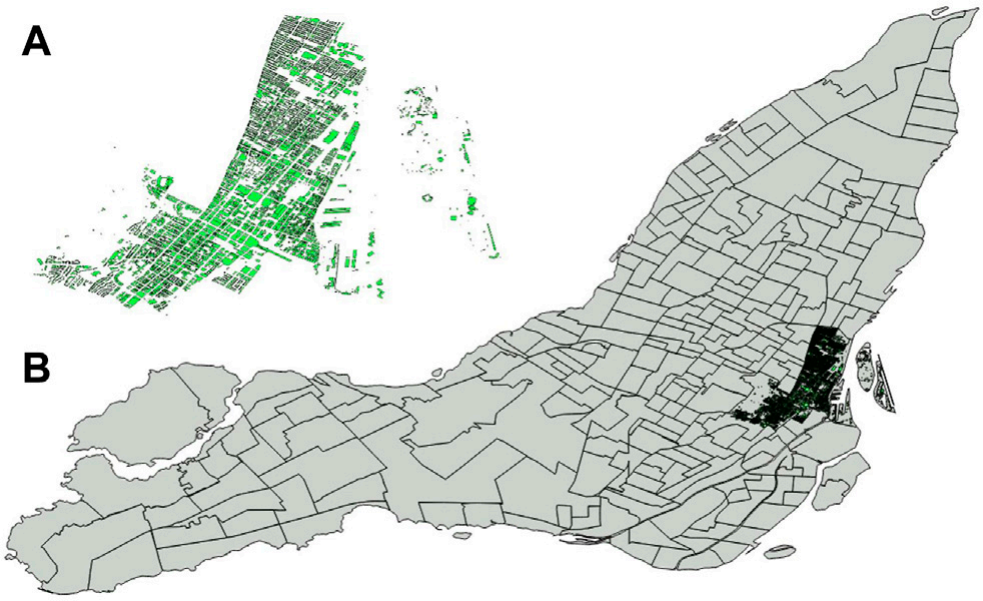
Neighborhood boundaries of the Urban Agglomeration area of Montréal and Ville-Marie **(A)**, and the footprint area from the CityGML buildings **(B)** visualized with QGIS.

Urban and rural areas differ in population density, infrastructure, building types, and land use. In the frame of the first application of FEW data model development, the investigation was done for the borough Ville-Marie in Montréal, Canada. A 3D CityGML model of Ville-Marie having sufficient quality is available for the study area. Montréal is located in the transition zone between different climatic zones. Summers are short, hot, and humid, and winters can be frigid, snowy, and windy. Most of the city is located on the peninsula Île de Montréal and more than three quarters of it is surrounded by water. The Urban Agglomeration of Montréal is constituted by the 19 boroughs and 14 linked municipalities. As a primary input to the presented FEW based workflows, the last updated official CityGML (building) data of Montréal available on the Open Data Portal of Montréal City (https://donnees.montreal.ca/) is used. Since the entire Montréal model is not available as open data, but only a few boroughs, the borough of Ville-Marie is used here as an example use case. Figure 3 shows the Urban Agglomeration of Montréal and the location of the use case area of Ville-Marie. According to the latest Census from 2016, the total population was 1,942,044 in 2016, with an increase of 2.9% between 2011 (1,886,481) and 2016. In total, 939,112 private dwellings were occupied by usual residents (870,373), and a population density per square kilometer of 3889.8. Ville-Marie had a population of 89,170 in 2016, representing 4.6% of the total population. The number of private dwellings in Ville-Marie was 61,643, which represents a share of 6.7%.

**FIGURE 4:**
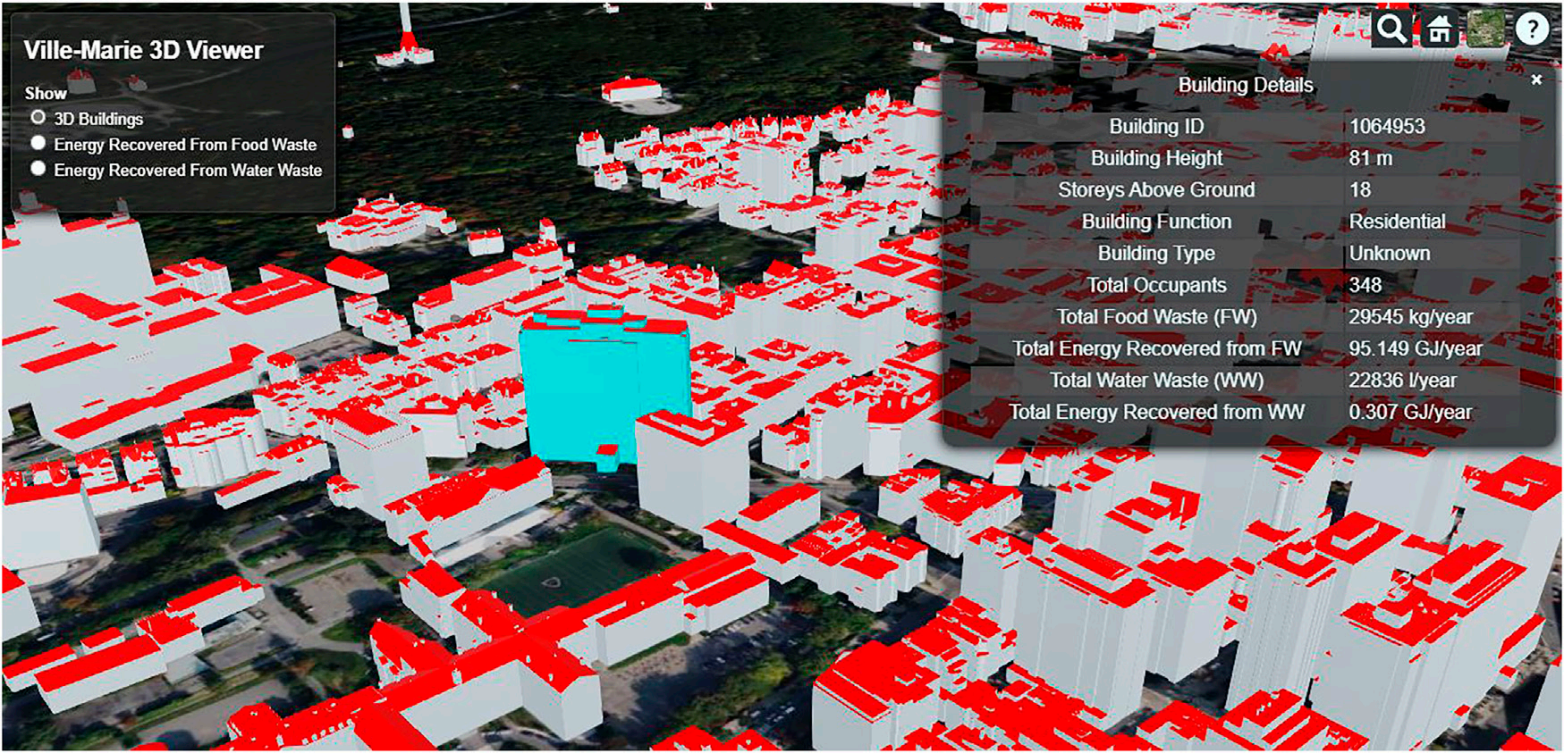
3D Visualization of some buildings in Ville-Marie (VM18_2016.gml) with Cesium.

The Ville-Marie area is divided into 18 separate CityGML files (VM01_2016.gml–VM18_2016.gml), and only geometrical data is available in the CityGML data. Figure 4 shows the 3D model visualized with the software Cesium (CesiumGS 2021) of the area of Ville-Marie. Hence any additional information on building functions and building occupancy are derived from OSM for each building. Layers extracted and used from OSM data are: office points, land use polygon, amenity polygon, amenity point, and amenity geometry.

### Waste-to-Energy From Food Waste

#### Estimation of Food Waste Generation

**FIGURE 5:**
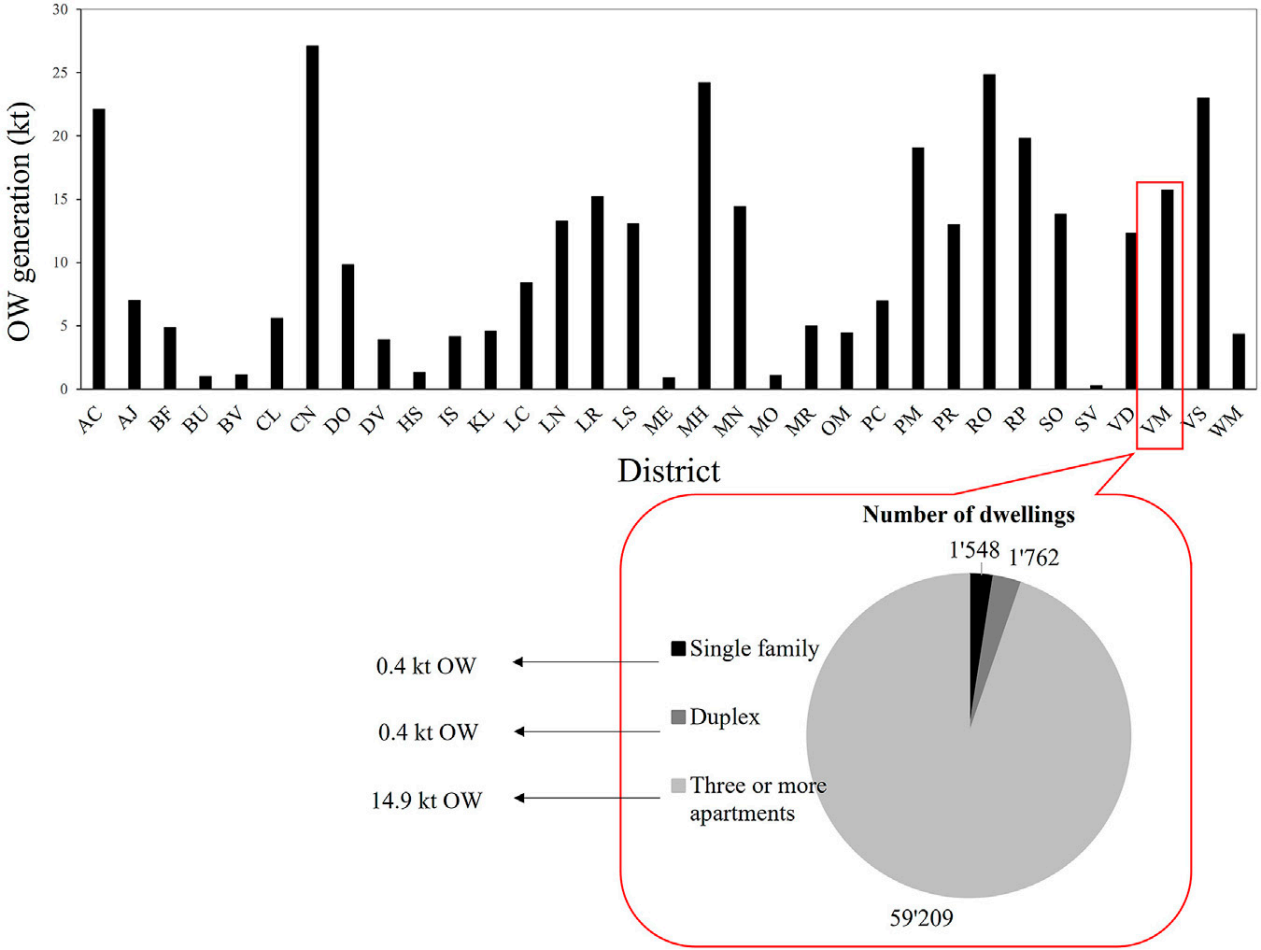
OW generation in Montréal’s districts including Ville-Marie in 2016 (Spreutels et al., 2019).

Malmir and Eicker (2020) did a statistical survey on waste flow based on the data of the Service de l′Environnement in Montréal. According to their study, 931 kt waste was generated in 2017, comprising 95% recyclables (OW, paper and cardboard, metal, glass, plastic, construction and demolition materials, textile, electronic waste and harmful household product) and 5% non-recyclables (non-recyclable construction and demolition and other materials). Figure 5 illustrates the generated OW in the districts of Montréal in 2016. The percentage of OW was assumed to be 40% of the total waste, including 22% FW, 36% yard waste, and 42% other OW (Malmir and Eicker 2020). According to this figure, 15.7 kt OW was generated in Ville-Marie district in Montréal in 2016. FW accounted for 3.46 kt from which 0.09, 0.10, and 3.28 kt OW was generated from 1,548 single family, 1,762 duplexes, and 59,209 three or more apartments, respectively (occupancies between 1.5 and 3.0 persons/dwelling) (Spreutels et al., 2019). According to Spreutels et al., 2019, and as illustrated in Figure 5, 15.7 kt OW was generated in Ville-Marie district in Montréal in 2016. The generated OW in this district is comparable to other districts of Montréal. For instance, Dorval (DV) and Ahuntsic-Cartierville (AC) generated 4 and 22 kt OW, respectively.

FW is not sorted efficiently everywhere, and it can be mixed by other waste. However, at the moment, there is little data on the actual generation of FW at the household level, and data for FW generation in the commercial sector are even scarcer (Massow et al., 2019). From 2002 to 2012, the amount of FW at the household level increased continuously in Canada. In 2002, the amount of FW produced per capita was about 58 kg, and in 2012, it reached already 85 kg (CEC 2017).

For calculating the FW generation, the equations presented by (Thiriet et al., 2020) were used as a starting point and were adapted so the CityGML files could be used as the primary input for the calculations.

The FW generated *Q*_*fw,res*_ per building in one year for a residential building type can be calculated with the following formula:

$$Q_{fw,res}=P_{res}\times q_{fw,res},$$ (2)

where *q*_*fw,res*_ is the annual FW generated per capita in kg. For the study area of Ville-Marie, a *q*_*fw,res*_ of 85 kg per capita and year was assumed. Sorted catering FW in commercial buildings (*Q*_*fw,com*_) occurs in school and health facility canteens and administrative and company canteens and can be calculated with the following formulas:

$$Q_{fw,com}=Nb_{meals}\times q_{meal}\times SSE_{s}.$$ (3)

*Nb*_*meals*_ is the number of meals served per year, *q*_*meal*_ is the FW generated per meal in kg and *SSE*_*s*_ source separation efficiency (without unit). The number of meals served in school canteens, health facilities, restaurants, and collective catering services is obtained from:

$$Nb_{meals,\;schools}=P_{stundents}\times Nb_{meals,student}\times Nb_{days,school},$$ (4)

$$Nb_{meals,hospitals,patients}=P_{patients}\times Nb_{meals,patient}\times Nb_{days,opening},$$ (5)

$$Nb_{meals,hospitals,employees}=P_{employees,hospital}\times Nb_{meals,employee}\times Nb_{days,working},$$ (6)

$$Nb_{meals,health,patients}=C_{capacity}\times Nb_{meals,day}\times Nb_{buisness\;days,\;week}\times Nb_{weeks,year},$$ (7)

$$Nb_{meals,restaurants}=P_{employees,restaurant}\times Nb_{meals\;served\;per\;employee},$$ (8)

where *P*_*student*_, *P*_*patients*_, *P*_*employees*_ is the number of students, patients or employees, *Nb*_*meal*_ is the number of meals per student, patients or employee in a day and *Nb*_*days*_ the number of days of school, number of opening days or number of working days per year.

#### Energy Recovery Potential From Biogas Produced With Food Waste

According to (ICF Consulting 2001), and based on the assumption of anaerobic digestion yield of CH_4_ at 0.220 m^3^/kg Volatile Solids, the biogas yield of FW is 0.113 m^3^/kg. Hence, the biogas yield of FW for the case study is 11.3 m^3^ per capita per year, leading to 1,003,429 m^3^ per year in Ville-Marie. The total amount of biogas produced is calculated as (Haight 2004):

$$V_{gas,produced}=\sum_{i=1}^{n}M\left(i\right)\times V_{gas}\left(i\right),$$ (9)

where, *V*_*gas, produced*_ is the total volume of biogas produced expressed in m³, *n* is the number of material components in the waste stream (here *n* = 1 because we only considered FW), *M*(*i*) is the mass of material component (*i*) in waste stream entering the digester, *V*_*gas*_(*i*) is the volume of biogas yielded per waste material component (*i*) expressed as m^3^/kg of material feed. As mentioned, the default biogas yield of FW is 0.113 m^3^/kg. Biogas produced in an anaerobic digestion facility is collected, treated to remove moisture, and then burned to produce electricity and/or steam. To estimate energy production, the landfill biogas energy calculation procedures have been adopted to provide estimates for energy production within an anaerobic digestion facility as follow (Haight 2004):

$$E_{recovered,fw}=V_{gas,produced}\times Cl_{gas}\times r_{eff}\times e_{eff}.$$ (10)

*E*_*recovered*_ is the energy recovered from the biogas in GJ, *Cl*_*gas*_ is the heat content of biogas in GJ/m^3^, *r*_*eff*_ is the gas recovery efficiency (%), *e*_*eff*_ is the energy recovery efficiency (%). The anaerobic digestion module calculates the energy consumed by the processes, including the electricity needed to operate sorting equipment and de-watering apparatus and the energy consumed while maintaining proper operating temperatures within the digester. A default value of 22% was considered for the energy consumption rates. Therefore, if we assume co-generation of electricity and steam, 1,003,429 m^3^ biogas per year in Ville-Marie will generate 28,597 GJ energy. Subtracting 22% energy consumption rates from this amount will lead to 22,306 GJ energy from FW. To compare, the end-use energy demand in Québec with around 8 million population was 1,770 petajoules in 2017.^1^

### Wastewater Generation and Wastewater Treatment

#### Estimation of Wastewater Generation

Schilling and Tränckner (2020) calculated the wastewater discharges at a high spatial resolution based on OpenStreetMap (OSM) data, combined with a dataset of the German official topographic–cartographic Information System (ATKIS), to estimate the volume of wastewater on a building level. Comparing the calculated daily values with inflow at pumping stations and sewage treatment plants for dry weather conditions showed that the method could generate realistic results. In this study, the equations from (Schilling and Tränckner 2020) were adapted so the CityGML files could be used as the main input for the calculations. The amount of discharged wastewater *Q*_*ww,res*_ for a residential building per year can be calculated with the following formula:

$$Q_{ww,res}=P_{res}\times q_{ww,res}\;\times \;f_{ww,res},$$ (11)

where *q*_*ww,res*_ is the discharge rates for a residential building in liter per person and year, *P*_*res*_ the number of people living in the building. The daily discharge rate per person in 2003 was around 225 iter, which results in an annual total of 82.125 m^3^ per person (see Table 3). Due to human activities such as cooking, drinking, etc., a part of the total consumed water does not end up in the wastewater stream; this effect is taken into account by *f*_*ww,res*_. The amount of wastewater is generally accounts, 75–80% of the water supplied.

**TABLE 3:** Water consumption per activity sector for Montréal (Source: Financement de l’eau, Document d’orientation, Ville de Montréal, 19 November 2003).

	Daily consumption in million cubic meters	Percentage of total consumption (%)	Daily consumption per person in L
Residential consumption	147	20	225
CII consumption (commercial, industrial and institutional)	239	33	363
Leaks and municipal use	339	47	516
Total	725	100	1,104

The discharge rate of wastewater for a commercial *Q*_*ww,com*_ or industrial *Q*_*ww, ind*_ building can be calculated with the following formulas:

$$Q_{ww,com}=A_{com}\times q_{ww,com}\;\times \;f_{ww,com},$$ (12)

$$Q_{ww,ind}=A_{ind}\times q_{ww,ind}\;\times \;f_{ww,ind},$$ (13)

where *q*_*ww,com*_ and *q*_*ww,ind*_ are the discharge rates of wastewater for a commercial and industrial building in m^3^ per m^2^ and year, related to the total useful building floor area. Since no distinction is made between commercial and industrial water consumption in Montréal, a value of 1.59 m^3^/m^2^ and year is used for both areas.

#### Energy for Wastewater Treatment Options

Raw municipal sewage is treated chemically, physically, and biologically in wastewater treatment processes. Before reuse or disposal, sludge must be treated. The treated sludge is then referred to as biosolids. Biosolids carry high water content and usually are de-watered prior to further treatment or disposal. In anaerobic digestion processes, microorganism's break down the organic matter in the sludge, and this accrue in the absence of oxygen, and by-products are methane-containing biogas and biosolids. Biogas produced from anaerobic digestion is a possible fuel source for digester heating or electricity generation. Biogas, besides methane, contains water vapor and small amounts of hydrogen sulfide and siloxanes, which must be removed before the biogas can be used as a fuel for electricity generation to prevent damage to the generation equipment. Electricity generation using biogas from anaerobic digestion varies depending on the generation of technology employed. Research from Burton and the Electric Power Research Institute (EPRI) shows that anaerobic digestion with biogas utilization can produce about 350 kWh of electricity for every million gallons (1 gallon = 3.78 L) of wastewater treated at the plant. Based on Clean Watershed Needs Survey (CWNS) data and biogas energy factors reported by Burton and EPRI the energy recovery potential for wastewater treatment plants using AD with biogas utilization was calculated using the Equation below.

$$Q_{ww,flow}=Q_{ww,res}+Q_{ww,com}+Q_{ww,ind},$$ (14)

$$ER_{recovered,ww}=Q_{ww,flow}\times BEF,$$ (15)

*ER*_*anaerobic*_: indicates the energy recovered from anaerobic digestion in kWh per year, *Q*_*ww,flow*_ the wastewater flow rate in m³ per year, and *BEF* the biogas energy. Reported biogas energy factors range from 0.0925 to 0.139 kWh/m^3^ for treated wastewater flows greater than 19.000 m^3^ per day (Stillwell et al., 2010). ion gallons per day.

## Results

In order to be able to check or validate the simulation results, calculated values were compared with ground truth data from official sources like census data and survey on waste flow based on the data of the Service de l′Environnement in Montréal. The calculation of the total residents for Ville-Marie results in a population of 72,606. In comparison, the statistical value for 2016 was 89,170, which corresponds to a deviation of 15.3%. A detailed review of the dataset showed that approximately 17% of the buildings could not be assigned a usage from the OSM data set due to missing OSM data, which corresponds to the buildings in the group Other (see Table 4). This means that approximately 23.9% of the area was not taken into account in the calculation. Considering this, it can be assumed that the calculation of the number of residents provides a realistic estimation. Furthermore, a number of about 15,744 people were calculated to be present in the commercial sector, which corresponds to a share of about 21.6% of the calculated population in this area. At present, it is not possible to determine whether the people who are present in the commercial sector actually live in this area.

**TABLE 4:** Summary of the building types and calculated useful floor area calculated.

Header	Residential	Commercial	Industrial	Other	Sum
Number of buildings	5,925	969	53	1,418	8,365
Total useful floor area in m^2^	4,356,388	6,184,718	884,065	3,587,201	15,012,371
Number of buildings in %	70.8%	11.6%	0.6%	17.0%	—
Total useful floor area in %	29.0%	41.2%	5.9%	23.9%	—

Table 5 shows the summary of the calculation results for FW and wastewater generation for the study area of Ville-Marie per building type. The energy recovery potential of FW is about 24,024 GJ/year, and that of wastewater is about 1,629 GJ/year, adding up to 25,653 GJ/year. Concerning the number of inhabitants, the energy recovery potential from anaerobic digestion of FW is 273.7 kWh/year in the residential sector. In the commercial sector, the energy recovery potential lies at 263.5 kWh/year per student, patient, or employee. Relating values to the calculated number of inhabitants in Ville-Marie results in 330.9 kWh/year for FW and 22.4 kWh/year for wastewater. It is further visible that the most significant potential (94%) for biogas production from anaerobic digestion lies in the collection of FW in the residential sector.

**TABLE 5:** Summary of the results for FW and wastewater generation.

	Residential	Commercial	Industrial	Sum
FW generation in kg/year	6,171,549	1,288,307	—	7,459,857
Total volume of biogas produced from FW in m^3^/year	697,385	145,579	—	842,964
Energy recovery potential from FW in GJ/year	19,875	4,149	—	24,024
Energy from FW per capita, student, patient or employee in kWh/year	273.7	263.5	—	537.3
Wastewater discharge rates in m^3^/year	4,770,244	7,866,961	1,405,664	14,042,869
Energy recovery potential from anaerobic digestion of wastewater in GJ/year	553	913	163	1,629
Energy recovered from anaerobic digestion of wastewater per capita, student, patient or employee in kWh/year	7.6	58.0	—	65.6
Energy recovery potential from FW and wastewater in GJ/year	20,429	5,062	163	25,653
Ratio of biogas potential from FW	97%	82%	0%	94%
Ratio of biogas potential from wastewater	3%	18%	100%	6%

**FIGURE 6:**
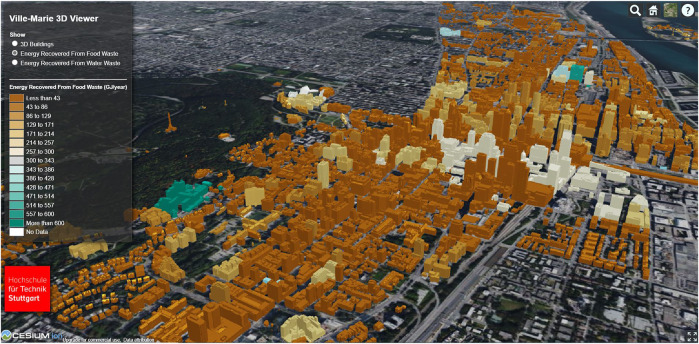
Visualization of the energy recovery potential from FW with Cesium, for some buildings in Ville-Marie, units of the legend—GJ/year.

**FIGURE 7:**
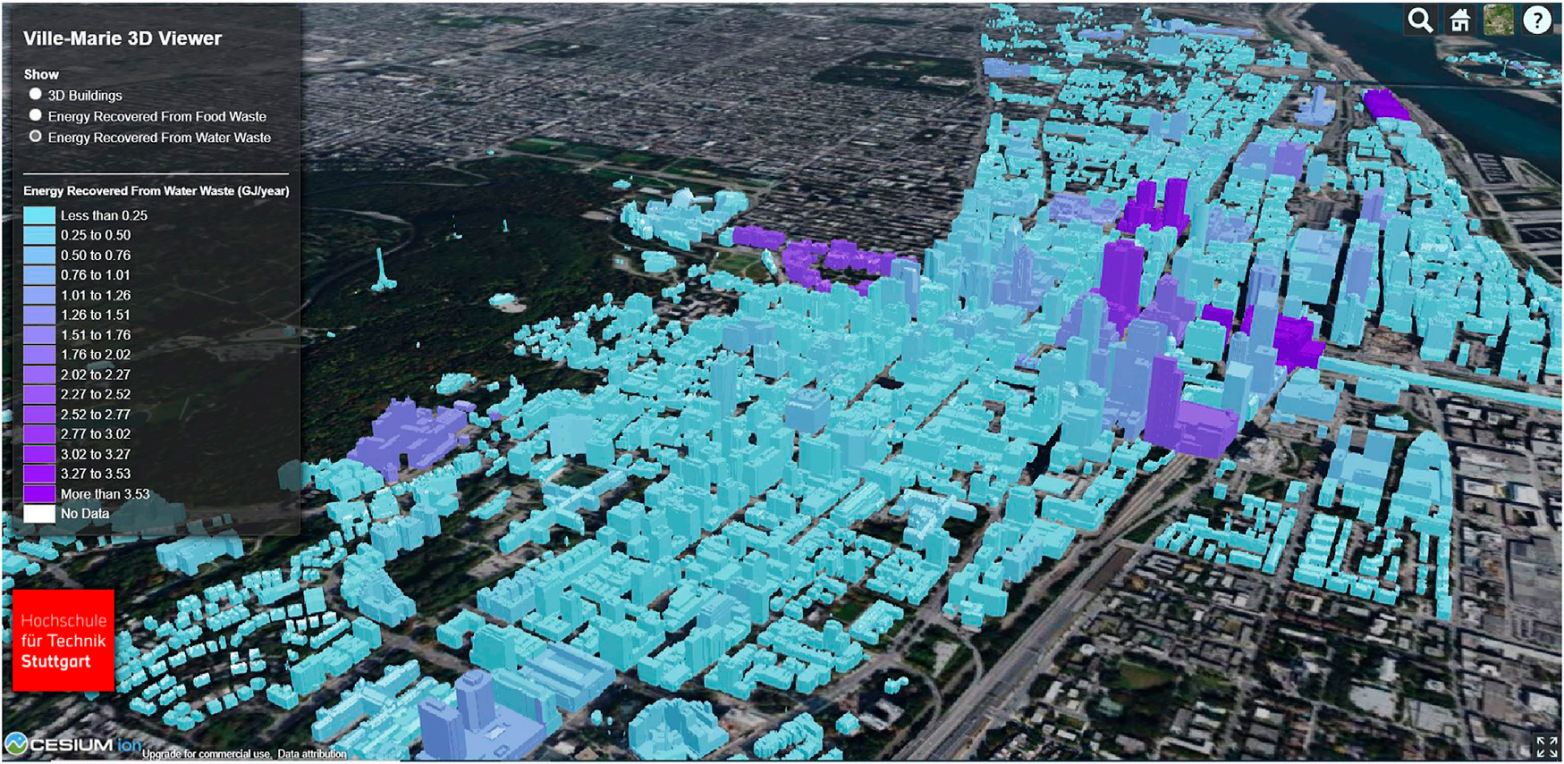
Visualization of the energy recovery potential from wastewater with Cesium for some buildings in Ville-Marie, units of the legend—GJ/year.

Figure 6 shows the generated color map for the energy recovery potential from FW for the area of Ville-Marie. It can be seen that several buildings have a particularly high potential for energy recovery from FW. After an extensive review of the data set, it was found that these are predominantly buildings from the group hospital. Buildings for which not all information could be determined to execute the calculations are shown in white. Figure 7 shows the energy recovery potential from the wastewater area; again, hospitals are identified.

## Summary

This study aimed to develop a workflow to calculate the amount of FW in the residential and commercial sectors and the wastewater generation from different building types based on the input of a 3D CityGML model. Furthermore, it aimed to provide information on extending the CityGML data model by developing a FEW ADE to support future FEW simulation workflows. The advantage of such an approach lies in its scalability; by considering FW and wastewater generation at the building level, it is possible to develop targeted scenarios that take local conditions into account. This makes it possible to introduce targeted local measures and to demonstrate their effect on the city level. The challenge in the development of such a workflow is, on the one hand, to bring together the different research domains from GIS, food, water, energy and, on the other hand, in the complexity of the available data sets. Data needed for the calculation are often not available as openly accessible data, or their granularity is not sufficient enough to be able to scale it down to the building level. In order to be able to scale down to the building level, it was necessary to create own libraries that represent the building stock and the building use. Relevant parameters such as the amount of wastewater generation in the individual categories (residential, commercial, and industrial) could be calculated using data sources from the City of Montréal. Comparing the calculated population with data from statistical surveys shows that the calculations provide a realistic estimate. Through the workflow designed in this study, it was possible to show which parameters can be relevant to develop a data model that can be transferred into a CityGML based FEW ADE. However, currently, there are still hurdles using the CityGML model provided by the city of Montréal as a basis for automated analysis of the FEW nexus at the city level. The used model did not have any information other than geometry information, so the dataset had to be enriched with further information from OSM in an effortful process. However, it is essential to mention that calculations with a strong dependency on user behavior, such as the FW and wastewater generation, can vary significantly from region to region. Often, data from the literature cannot be transferred from one region to the other. Uncertainties arise in calculating the number of people present in the buildings, but the additional information of the building geometry, especially the height and volume data, allows a realistic estimation. This also shows the advantage of using 3D city models compared to 2D GIS data. In the next steps, it should be examined to which extent the workflow can be used for city planning activities, and it should also be examined whether the workflow can be applied to other cities with the same structure in population, building typology, etc. and in which parts adjustments need to be made in the libraries. Furthermore, more investigation is needed to estimate existing errors in the building geometry on the domain-specific calculations such as FW and WW generation. A good indicator of whether a building model can be used for the calculation is the value of *R*_*GML*_ (the ratio of envelope area and building volume calculated from the GML data). Buildings with a value above 1 can be considered significant geometry problems and can lead to a high deviation between calculation and realistic values. For the area of Ville-Marie, around 200 buildings have a high value of *R*_*GML*_.

## Data Availability

The original contributions presented in the study are included in the article/Supplementary Material, further inquiries can be directed to the corresponding author.

## Footnotes

1
https://www.cer-rec.gc.ca/en/data-analysis/energy-markets/provincial-territorial-energy-profiles/provincial-territorial-energy-profiles-quebec.html.
