# Supplementary material for: Using 3D CityGML for the Modeling of the Food Waste and Wastewater Generation—A Case Study for the City of Montréal
Source: Front Big Data. 2021 Jun 22;4:662011. doi: 10.3389/fdata.2021.662011 (PMC8257941; doi:10.3389/fdata.2021.662011)
Supplement: Supplementary file 1 [file DataSheet1.docx]

Supplementary Material

# Supplementary Figures, Tables and Equations

**The factor *R_cube,GML_***

$R_{cube,GML}=\frac{\frac{A_{envelope,GML}}{A_{gr}\times h_{GML}}}{R_{GML}}$ (1)

where *h_GML_* is the building height taken from the CityGML model, *A_envelope,GML_* the building envelope area calculated from the GML data in m², *A_gr_* the ground area (building footprint) of the building from the CityGML model, *R_GML_* the ratio of envelope area and building volume calculated from the GML data.

**Estimation of the typical floor heights for the building types**

To determine the typical floor heights of the individual building types, buildings were randomly selected from the 3D model, and the information on the total height was noted. The next step was to check whether the building type determined from the OpenStreetMap data was realistic. Finally, the number of stories of the selected building was determined via Google Street View. Table 1 illustrates an example data set of two buildings created for the area of Ville-Marie, and Table 2 shows the determined average floor heights for the different building types. The floor height of the type other represents the mean value of the other three types.

Table 1. Example data set of two buildings

| **3D Model** | **3D Model** | **Google Maps top view** | **Google Street View view on the facade** |
| --- | --- | --- | --- |
| gml_id = 1179741, type = commercial, citygml measured height = 25.9 m, detemined stories above ground 5, resulting floor height = 6.475 | | | |
| 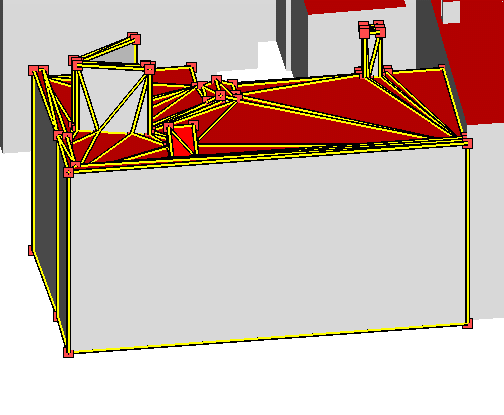 | 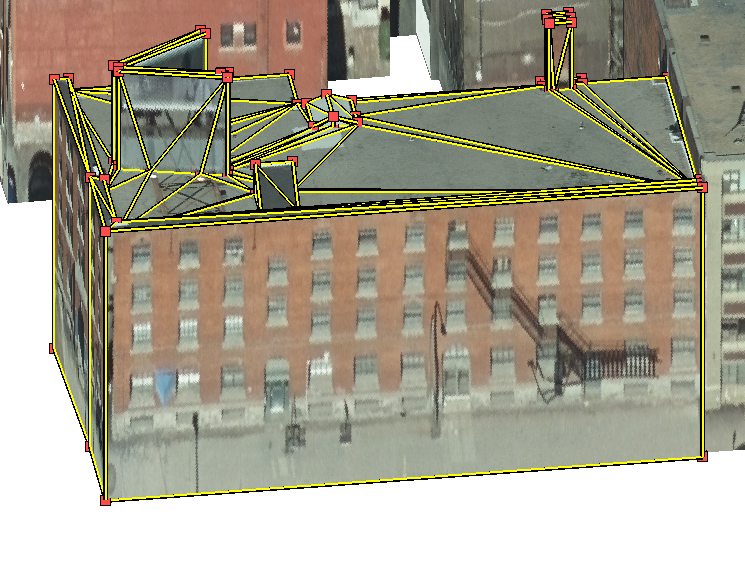 | 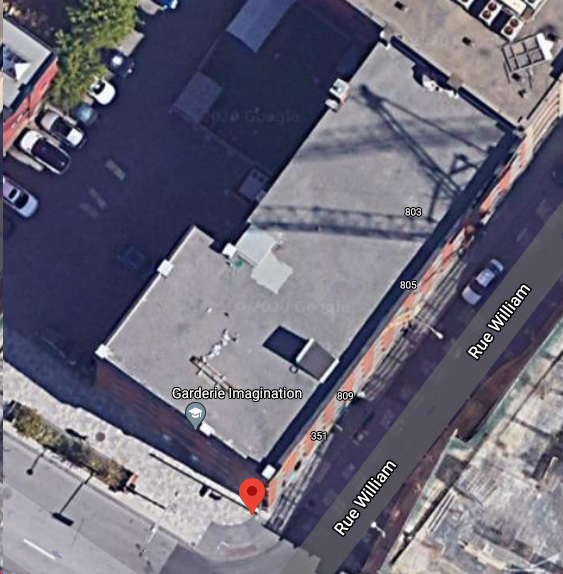 | 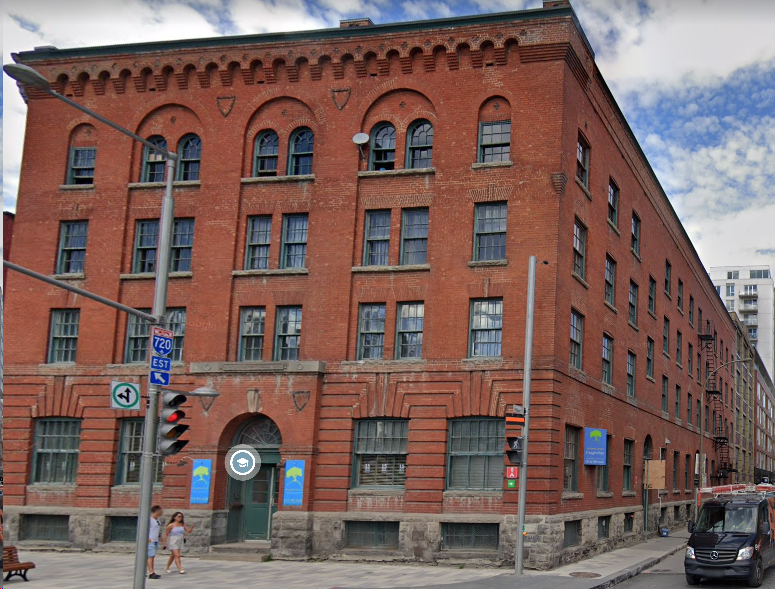 |
| gml_id = PC-36575, type = residential, citygml measured height = 48.8 m, detemined stories above ground 13, resulting floor height = 3.75 | | | |
| 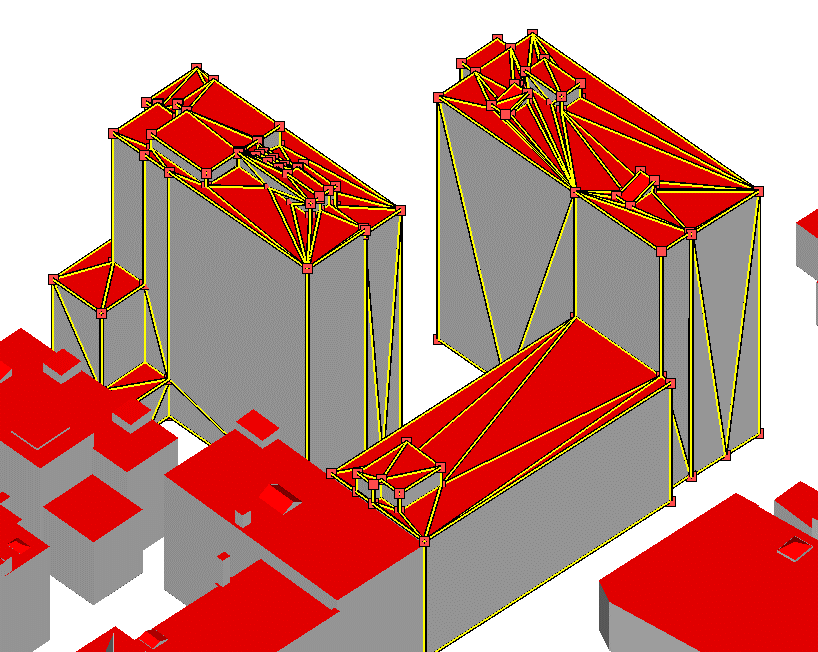 | 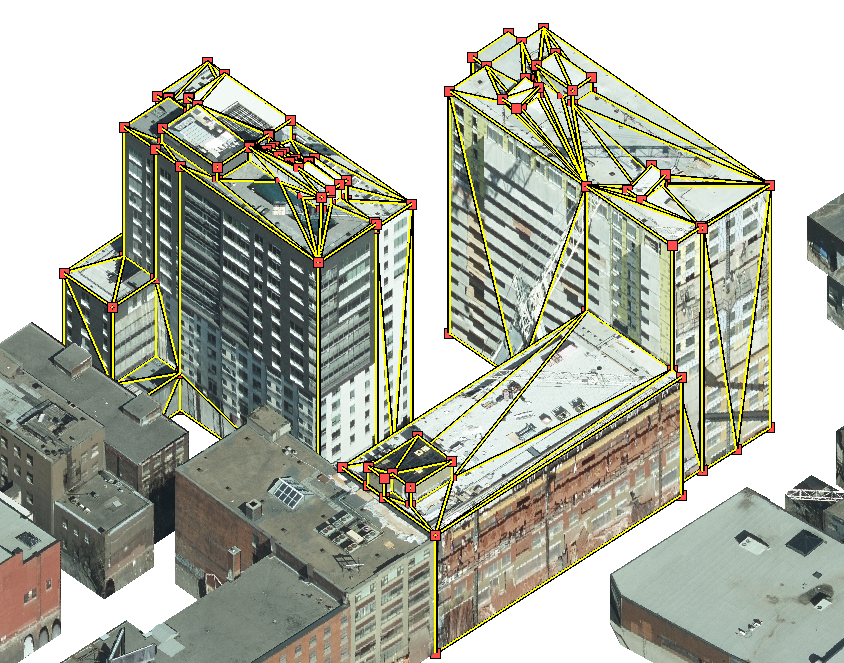 | 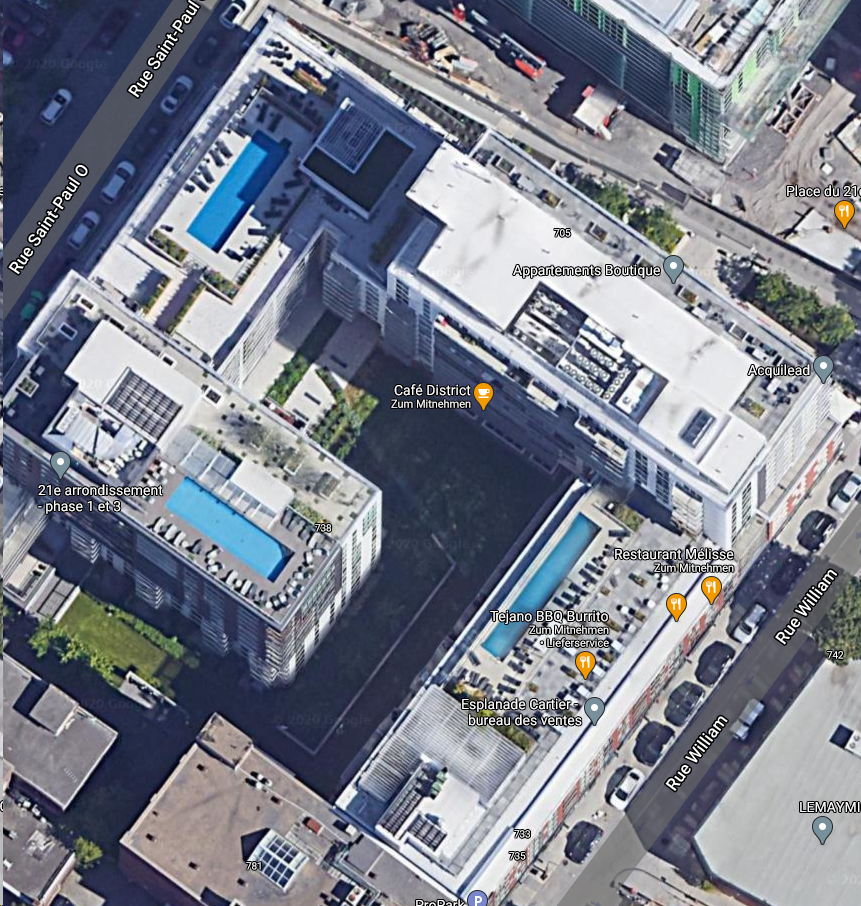 | 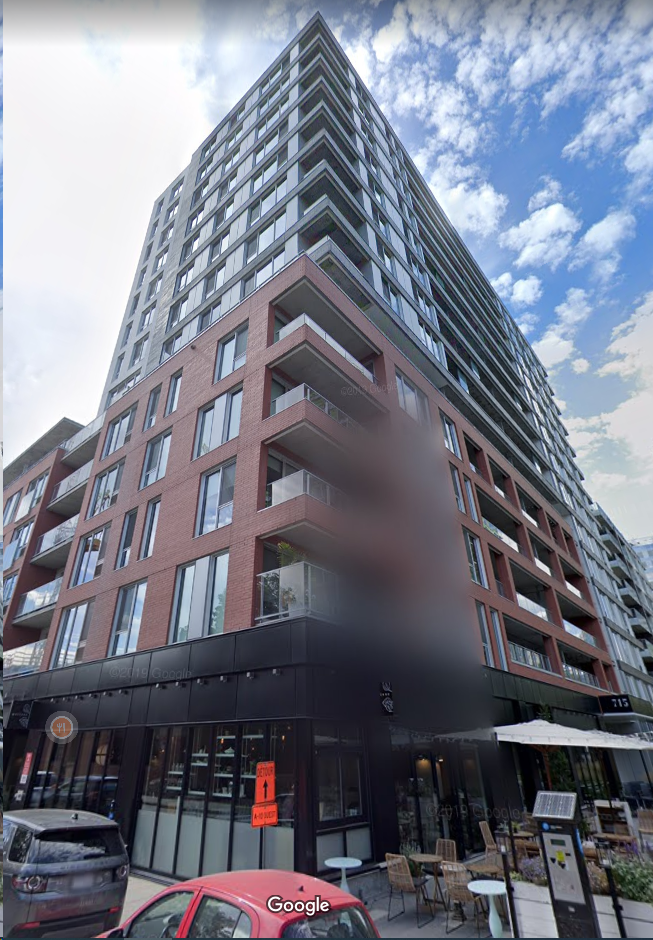 |

Table 2. Typical floor heights in Montréal per building type

| **Building type** | **Floor height in m** |
| --- | --- |
| Residential | 4.46 |
| Commercial | 5.97 |
| Industrial | 5.97 |
| Other | 5.47 |

Table 3. Geometric validation of the CityGML data set provided by the city of Montréal with the Software CityDoctor2 - version 3.8.0 (Beuth Hochschule für Technik Berlin 2021)

| **CityDoctor Validation** | **Total Buildings** | **Buildings with errors** | **unalerted buildings** | **Ratio buildings with errors** |
| --- | --- | --- | --- | --- |
| VM01_2016.gml | 330 | 58 | 272 | 17,6% |
| VM02_2016.gml | 104 | 71 | 33 | 68,3% |
| VM03_2016.gml | 344 | 100 | 244 | 29,1% |
| VM04_2016.gml | 250 | 56 | 194 | 22,4% |
| VM05_2016.gml | 259 | 34 | 225 | 13,1% |
| VM06_2016.gml | 234 | 69 | 165 | 29,5% |
| VM07_2016.gml | 560 | 182 | 378 | 32,5% |
| VM08_2016.gml | 136 | 23 | 113 | 16,9% |
| VM09_2016.gml | 581 | 98 | 483 | 16,9% |
| VM10_2016.gml | 435 | 196 | 239 | 45,1% |
| VM11_2016.gml | 542 | 129 | 413 | 23,8% |
| VM12_2016.gml | 171 | 43 | 128 | 25,1% |
| VM13_2016.gml | 834 | 128 | 706 | 15,3% |
| VM14_2016.gml | 505 | 53 | 452 | 10,5% |
| VM15_2016.gml | 968 | 93 | 875 | 9,6% |
| VM16_2016.gml | 968 | 113 | 855 | 11,7% |
| VM17_2016.gml | 843 | 67 | 776 | 7,9% |
| VM18_2016.gml | 307 | 116 | 191 | 37,8% |
| **Total** | **8371** | **1629** | **6742** | **19,5%** |

Table 4. Distribution of water consumption in a house in Montréal (Source: Financement de l’eau, Document d’orientation, Ville de Montréal, 19 November 2003)

| **Type of use** | **Percentage of total consumption** | **Proportion for a consumption of 225 liters per person** | **Proportion for a consumption of 350 liters per person** |
| --- | --- | --- | --- |
| Exterior maintenance (watering, pool, etc.) | 30% | 68 | 105 |
| Toilet (18 to 28 liters are used for each flush) | 30% | 68 | 105 |
| Washing (dishes and clothes) | 20% | 45 | 70 |
| Baths and showers | 19% | 42 | 66 |
| Food | 1% | 2 | 3 |
| Total | 100% | 225 | 350 |

**Data used to calculate the amount of FW in commercial buildings**

Bawaneh et al. (2019) analyzed the end-use energy consumption data in healthcare systems for hospitals in the United States for different climate zones. Table 5 represents the operational details per climate zone. Based on the findings from (Bawaneh et al. 2019), the floor space per worker (employee) and the floor space per bed (patient) were calculated and used for the simulation of the resulting FW in hospitals.

Table 5. Hospitals operational details per climate zone in the United States (Bawaneh et al. 2019), calculation of the floor space per worker/bed own calculation.

|  | Number of Buildings | Total Floorspace (Million m²) | Total Workers (Thousands) | Total Patient Beds (Thousands) | Floor Space per Worker (m²/worker) | Floor Space per Bed (m²/bed) |
| --- | --- | --- | --- | --- | --- | --- |
| Total | 3040 | 181.9 | 3340 | 915 | 54.5 | 198.8 |
| Zone 1 | 517 | 30.9 | 628 | 165 | 49.2 | 187.3 |
| Zone 2 | 818 | 47.3 | 803 | 241 | 58.9 | 196.3 |
| Zone 3 | 501 | 23 | 529 | 132 | 43.5 | 174.2 |
| Zone 4 | 794 | 47.1 | 875 | 251 | 53.8 | 187.6 |
| Zone 5 | 410 | 33.6 | 505 | 127 | 66.5 | 264.6 |

The values for the FW generated per meal for the patients and employees in a hospital and the FW generated in health facilities were obtained from (Strotmann et al. 2017). The following assumptions were made in this regard:

- Patients in a hospital receive three meals a day, and no distinction is made between breakfast, lunch, and dinner. An average value of the three meals is used for the calculation.
- Every employee in a hospital goes to the canteen once a day
- the FW in retirement homes are in a similar magnitude as the FW for other health facilities

Table 6. Daily plate waste per person in a hospital and retirement home in g (Strotmann et al. 2017)

|  | Measurement 1 | Measurement 2* | Mean value | Reduction in % |
| --- | --- | --- | --- | --- |
| Hospital (patients) | 385 | 318 | 352 | 17 |
| Cafeteria (employees) | 36 | 18 | 27 | 50 |
| Retirement Home | 202 | 152 | 177 | 25 |

* After implementing reduction measures in the five categories: information, communication, products, processes, and customer needs.

**Data used to calculate the amount of energy recovered from FW**

Table 7. Biogas data (Haight 2004)

| **Parameter** | **Value** |
| --- | --- |
| Biogas composition |  |
| - CH_4_ | 55% |
| - CO_2_ | 45% |
| Heat content of biogas GJ/m³ | 0.038 |
| Default gas recovery efficiency | 100% (assumes no leakage) |
| Default energy recovery efficiency |  |
| - Production of electricity | 20% |
| - Production of steam | 70% |
| - Co-generation of electricity and steam | 75% |

**Table 8. Parameters used for the calculation**

|  |  | **Value** | **Unit** | **Source** |
| --- | --- | --- | --- | --- |
| **Food waste calculation** |  |  |  |  |
| **Residential buildings** |  |  |  |  |
| Factor that takes into account the actual usable floor space in residential buildings | *f_usage,res_* | *0.75* | *-* | *estimation* |
| Average area used per person in this region for living | *A_P,res_* | *60* | *m²/person* | *estimation* |
| FW generated per person | *q_fw,res_* | *85* | *kg/(capita*year)* | (CEC 2017) |
| **Schools** |  |  |  |  |
| Factor that takes into account the actual usable floor space in schools | *f_usage,school_* | 0.75 | - | estimation |
| FW generated per meal | *q_meal,school_* | 0.1 | kg/meal | estimation, based on numbers found for hospitals and health facilities |
| Source separation efficiency | *SSEs* | 0.9 | - | estimation |
| Average area used per student in that region | *A_P,student_* | 35 | m²/student | estimation |
| Number of days of school per year | *Nb_days of school_* | 260 | days/year | estimation |
| Number of meals served per student and day | *Nb_meals,student_* | 2 | meals/(student*day) | estimation |
| **Hospitals** |  |  |  |  |
| Factor that takes into account the actual usable floor space in hospitals | *f_usage,hospital_* | 0.75 | - | estimation |
| Average area used per patient in a hospital | *A_P,patient,hospital_* | 198.8 | m²/patient | (Bawaneh et al. 2019) |
| Number of opening days in a hospital per year | *Nb_days,opening,hospital_* | 365 | days/year | estimation |
| Number of meals served per patient and day | *Nb_meals,patient_* | 3 | meals/(patient*day) | estimation |
| FW generated per meal and patient in a hospital | *q_meal,hospital,patient_* | 0.385 | kg/meal | (Strotmann et al. 2017) |
| Average area used per employee in a hospital | *A_P,employee,hospital_* | 54.5 | m²/employee | (Bawaneh et al. 2019) |
| Number of working days | *Nb_days,working,hospital_* | 260 | days/year |  |
| Number of meals served per employee and day | *Nb_meals,employee_* | 1 | meals/(employee*day) | estimation |
| FW generated per meal and employee in a hospital | *q_meal,hospital,employee_* | 0.036 | kg/meal | (Strotmann et al. 2017) |
| **Health facilities** |  |  |  |  |
| Average area used per patient in a health facility in that region | *A_P,patient,health_* | 150 | m²/patient | estimation |
| Number of opening days of a health facility | *Nb_days,opening,health_* | 320 | days/year | estimation |
| Number of meals served per patient and day in a health facility | *Nb_meals,health,patient_* | 3 | meals/(patient*day) | estimation |
| FW generated per meal and patient in a health facility (retirement home) | *q_meal,health,patient_* | 0.202 | kg/meal | (Strotmann et al. 2017) |
| **Restaurants** |  |  |  |  |
| Factor that takes into account the actual usable floor space in restaurants | f_usage,restaurants_ | 0.75 | - | estimation |
| Average area used per employee in a resaurant in that region | A_P,employee,restaurant_ | 100 | m²/employee | estimation |
| Number of meals served per employee in one year | Nb_meals,employee,restaurant_ | 2920 | meals/(employee*year) | (Thiriet et al. 2020) |
| FW generated per meal and customer in a restaurant | q_meal,restaurant,customer_ | 0.109 | kg/meal | (McAdams et al. 2019) |
| **Biogas production** |  |  |  |  |
| The volume of biogas yielded per waste material component (i) | V_gas_ | 0.113 | m³/kg | (ICF Consulting 2001) |
| Heat content of biogas in GJ/m³ | Cl_gas_ | 0.038 | GJ/m³ | (Haight 2004) |
| Gas recovery efficiency | r_eff_ | 100 | % | estimation |
| Energy recovery efficiency | e_eff_ | 75 | % | estimation |
| **Wastewater discharges** |  |  |  |  |
| The discharge rates for a residential building | q_ww,res_ | 82.125 | m³/(person*year) | Data calculated based on information from the City of Montréal for the year 2003 |
| Discharge rate of wastewater for a commercial building | q_ww,com_ | 1.59 | m³/(m²*year) | Data calculated based on information from the City of Montréal for the year 2003 |
| Building footprint area of the commercial building | A_com_ | 0 | m² | Input from 3D Data, equal to the ground area of the building |
| Discharge rate of wastewater for an industrial building | q_ww,ind_ | 1.59 | m³/(m²*year) | Data calculated based on information from the City of Montréal for the year 2003 |
| Building footprint area of the commercial building | A_ind_ | 0 | m² | Input from 3D Data, equal to the ground area of the building |
| **Biogas production in WWT** |  |  |  |  |
| Biogas energy factor | BEF | 0.116 | kWh/m³ | Mean value form (Stillwell et al. 2010) (0.0925 - 0.139) |

References

Bawaneh K, Ghazi Nezami F, Rasheduzzaman M, Deken B. 2019. Energy Consumption Analysis and Characterization of Healthcare Facilities in the United States. [place unknown]: [publisher unknown] (Energies; vol. 12). ISBN: 1996-1073.

Beuth Hochschule für Technik Berlin. 2021. CityDoctor2. 3.8.0. Berlin: Free Software Foundation; [accessed 2021 Apr 5]. https://​projekt.beuth-hochschule.de​/​citydoctor2.

CEC. 2017. Characterization and Management of Organic Waste in North America—Foundational Report. Canada: Commission for Environmental Cooperation. www.cec.org.

Haight M. 2004. Techincal Report: Integrated Solid Waste Management Model. School of Planning University of Waterloo, Canada.

ICF Consulting. 2001. Determination of the Input of Waste Management Activities on Greenhouse Gas Emissions. Report submitted to Environment Canada.: 32 pages and Appendices and Tables.

McAdams B, Massow M von, Gallant M, Hayhoe M-A. 2019. A cross industry evaluation of food waste in restaurants. Journal of Foodservice Business Research. 22:449–466. doi:10.1080/15378020.2019.1637220.

Strotmann C, Friedrich S, Kreyenschmidt J, Teitscheid P, Ritter G. 2017. Comparing Food Provided and Wasted before and after Implementing Measures against Food Waste in Three Healthcare Food Service Facilities. [place unknown]: [publisher unknown] (Sustainability; vol. 9). ISBN: 2071-1050.

Thiriet P, Bioteau T, Tremier A. 2020. Optimization method to construct micro-anaerobic digesters networks for decentralized biowaste treatment in urban and peri-urban areas. Journal of Cleaner Production;243:118478. http://​www.sciencedirect.com​/​science/​article/​pii/​S0959652619333487. doi:10.1016/j.jclepro.2019.118478.
